# Federalism and Health System Performance: Lessons From Sub‐National Case Studies in Nepal

**DOI:** 10.1002/puh2.70439

**Published:** 2026-11-07

**Authors:** Deepak Paudel, Subheksha Adhikari, Ghanshyam Gautam, Yeshoda Aryal, Khim Bahadur Khadka, Sagar Prasad Ghimire, Sunita Dharel, Anil Bhattarai, Kimat Adhikari

**Affiliations:** ^1^ Center for International Health LMU Munich Munich Germany; ^2^ Community Health and Nutrition UNICEF Nepal Lalitpur Nepal; ^3^ Ministry of Health, Hetauda Bagmati Province Nepal; ^4^ HERD International Lalitpur Nepal; ^5^ Ministry of Health and Food Safety Kathmandu Nepal; ^6^ Ministry of Health, Pokhara Gandaki Province Nepal; ^7^ Bhimad Municipality, Bhimad Gandaki Province Nepal; ^8^ Nepal Health Economics Association Kathmandu Nepal; ^9^ World Health Organization, Pulchowk Lalitpur Nepal

**Keywords:** devolution, federalism, health system, Nepal, sub‐national

## Abstract

**Background:**

Nepal's sub‐national health systems face persistent capacity gaps requiring assessments beyond World Health Organization (WHO) building blocks to capture system performance, effectiveness, efficiency, and equity.

**Methods:**

This short communication synthesizes three case studies: (1) technical assistance evaluating WHO framework capacity gains in municipal learning sites (pre/post scores) in two municipalities; (2) participatory institutional capacity assessments across seven domains in 79 municipalities of Karnali Province; and (3) a mixed‐methods study using 166 household surveys, 12 key informant interviews, and one focus group discussion in a rural municipality of Bagmati Province applying extended WHO framework.

**Results:**

Targeted technical assistance yielded rapid municipal capacity gains, particularly in low‐baseline sites in Koshi and Madhesh; Karnali assessments showed legacy functions (supply, budgeting) outperforming new governance roles (policy, monitoring, and evaluation); rural municipality in Bagmati's moderate performance linked effectiveness, efficiency, and equity to geographic access barriers, staff shortages, gender biases, insurance gaps, and disaster impacts.

**Conclusions:**

Federalism's early phase demands urgent tier‐to‐tier collaboration and bottom‐up assessments incorporating community realities, gender, and social inclusion, which are not captured in the WHO's building blocks. Streamlined, harmonized support across federalized role division is essential to build responsive, people‐centered sub‐national health systems meeting Nepal's evolving demands.

## 1 Introduction

Following the promulgation of Nepal's [1] 2015 Constitution, the country adopted a federal governance system that devolved authority to seven provincial and 753 local governments (LGs) [2]. This restructuring delineated responsibilities between three tiers of government for planning, managing, and delivering services. The health system is defined by the World Health Organization (WHO) through six building blocks: service delivery, health workforce, health information systems, access to essential medicines, health financing, and leadership/governance [3, 4]. Although this framework provides a simple and structured lens, it has been criticized for oversimplifying the dynamic complexity of health systems by treating components as static and overlooking critical elements, such as community systems. In response, the concept of “beyond the building blocks” has emerged, emphasizing functions, relationships, and interconnected processes that enable systems to operate effectively [5].

Globally, health system devolution has been recognized for its potential to improve the responsiveness of health systems to local needs; however, experiences in Pakistan, Indonesia, Bangladesh, and India have produced mixed outcomes [6]. In Nepal, federalization generated expectations to improve health service delivery through stronger community engagement, bottom‐up planning, and enhanced accountability mechanisms. Sub‐national governments can strategically leverage federal resources to recalibrate their local priorities and restructure their systems [7]. Despite the early signs of progress, such as the development of locally tailored health policies, increased investment in infrastructure, regularity of service delivery, and expanded outreach services, concerns remain regarding institutional capacity, health system efficiency, and equitable access to quality health services.

Nearly a decade after federalization, the country stands at a critical stage as it continues to strengthen provincial and local health systems while striving to achieve the targets of universal health coverage (UHC) and the Sustainable Development Goals (SDGs) [8]. Nepal has recently revised SDG commitments, lowering mortality targets and delaying LDC graduation amid fiscal pressures [9]. Over the next decade, Nepal is likely to face reduced foreign aid and shifting donor engagement in the health sector [10].

Several reviews have assessed Nepal's health system capacities, current efforts, and areas for improvement [2, 11, 12]; however, evidence on how sub‐national systems have performed and evolved during the first decade of federalization is limited. Understanding shifts in health system performance, governance, and equity at the sub‐national level is critical for advancing Nepal's federal health agenda. This study examines Nepal's experience of devolving health systems under federalism through three case studies that represent diverse methods, timelines, and regions of the country. These case studies were selected through a national consultation process as promising examples. Importantly, they draw on and highlight the co‐authors’ direct involvement in designing and delivering technical assistance or assessing health systems.

## 2 Structure and Functions Within Nepal's Health System

At the federal level, the Ministry of Health and Food Safety (MoHFS, recently renamed from Ministry of Health and Population) provides overall policy direction, strategy, guidelines, standard formulation, regulation, financing, and coordination of national health programs, while also delivering specialized services, such as tertiary care and surveillance at points of entry. The provincial governments lead planning, implementation/operation, technical supervision, and management of provincial health institutions. LGs have become the primary implementers of basic health and sanitation services and oversee the operation of primary care facilities, including basic health service centers, health posts (HP), and primary hospitals.

The allocation of health responsibilities follows the constitutional division of powers outlined in the constitution of Nepal [1] and further elaborated by the Local Government Operation Act [13], the federal unbundling framework [14], the Public Health Services Act [15], and the Public Health Regulations [16]. These legal instruments have shifted technical, administrative, and financial authority closer to the communities, fostering greater local accountability and responsiveness in service delivery. LGs play a crucial role in contextual bottom‐up planning process, delivery of basic health services through peripheral health facilities, and functionalize of community health systems, including female community health volunteers (FCHVs), community health workers, and outreach clinics, as shown in Figure 1. This interface continues to provide the foundational link between households and health systems, as elaborated in Table 1.

**FIGURE 1:**
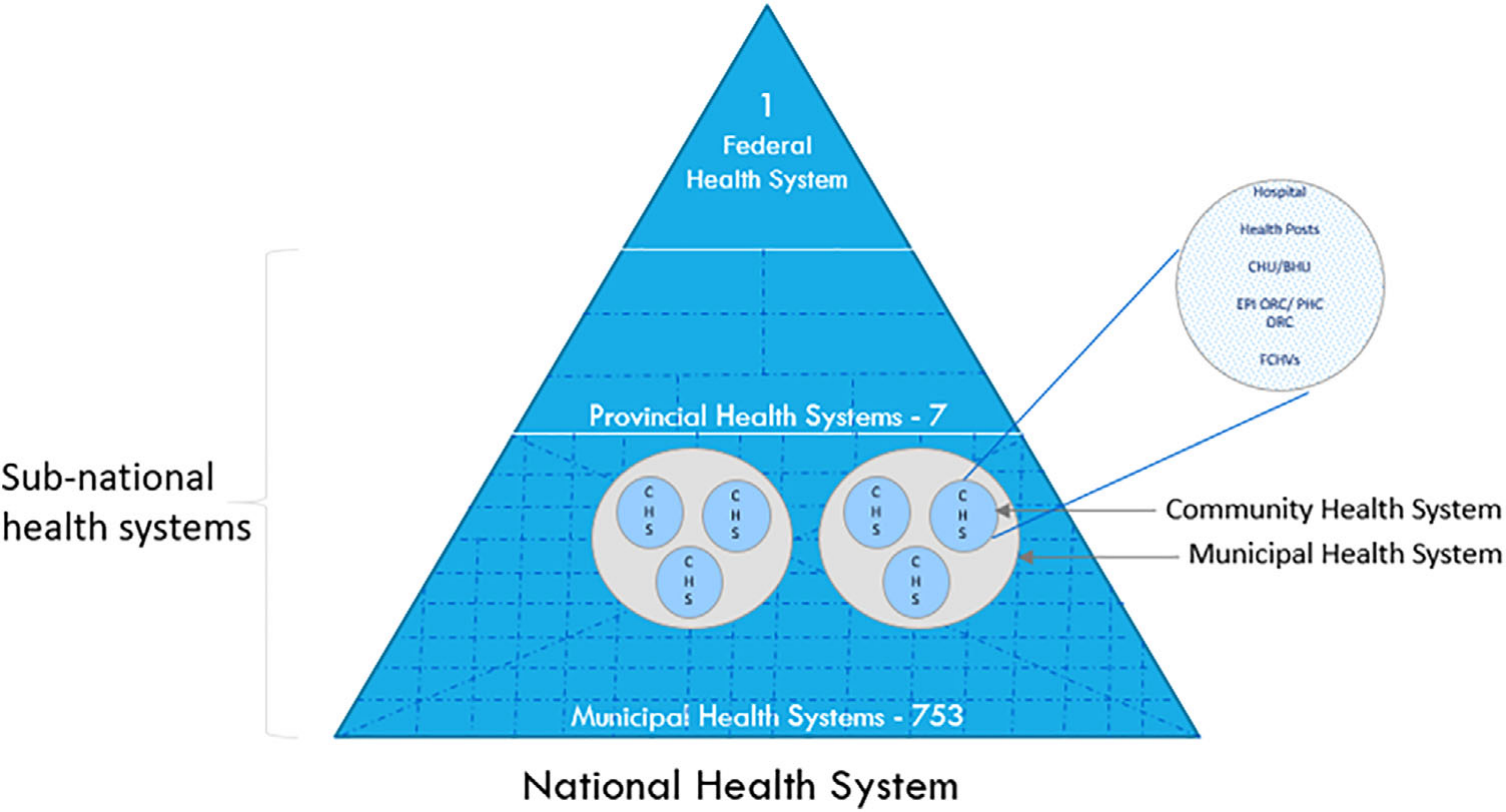
Organizational architecture of Nepal's federalized health systems, illustrating linkages between federal, provincial, municipal, and community levels. CHUs, community health units; FCHVs, female community health volunteers. *Source:* Author's own work.

**TABLE 1:** Structure and functions of Nepal's federal health system across government levels, including key institutions and staffing.

Level	Institutions	Key functions
Federal	Ministry 4 departments 6 councils 30 federal hospitals 28 specialized hospitals	Sets national policy, standards, laws, and regulations for the health system Oversees human resources, finance, planning, and M&E Delivers tertiary and specialized hospital care Manages intersectoral coordination, donor relations, global health security requirements, and monitoring commitments
Provincial	Ministry (7) Health directorates (7) Training/Logistics/Lab centers District health officers (77) Provincial hospitals (56)	Plans and monitors provincial‐level health programs Provides technical backstopping to municipalities and hospitals Delivers secondary and multispecialty care through provincial hospitals Regulates provincially delivered health services
Local	Municipality Health Sections/Divisions (753) Primary hospitals (25) Primary health care centers (196) Health posts (3806) Urban health centers/CHUs (289)	Plans and manages municipal‐level health activities focusing on basic health services Delivers primary care, such as family planning, immunization, outpatient diagnosis, minor illness treatment, and minor surgery Extends outreach to underserved populations Manages community health workers and community‐focused services

Abbreviation: CHUs, community health units.

## 3 Experiences of the Federalized Health System at the Sub‐National Level in Nepal

This study presents three cases illustrating sub‐national health system capacity and performance in Nepal during early federalization and nearly a decade later. Cases 1 and 2 document early implementation experiences, whereas Case 3 examines more recent sub‐national health system performance.

**Case 1**: The Nepal Health Sector Support Program (NHSSP), a technical assistance initiative funded by the British government, supported selected municipalities as the learning sites for understanding and improving municipal health systems. The health systems’ capacity was measured using an assessment tool tailored to the post‐federalized context of Nepal.

The assessment in 2018–19 in two LGs from Koshi and Madhesh Provinces revealed varied municipal capacities. Capacity gains were higher in the municipality with a lower baseline, whereas the one starting with stronger capacity showed only modest improvement in its overall score; see Figure 2a.

**FIGURE 2:**
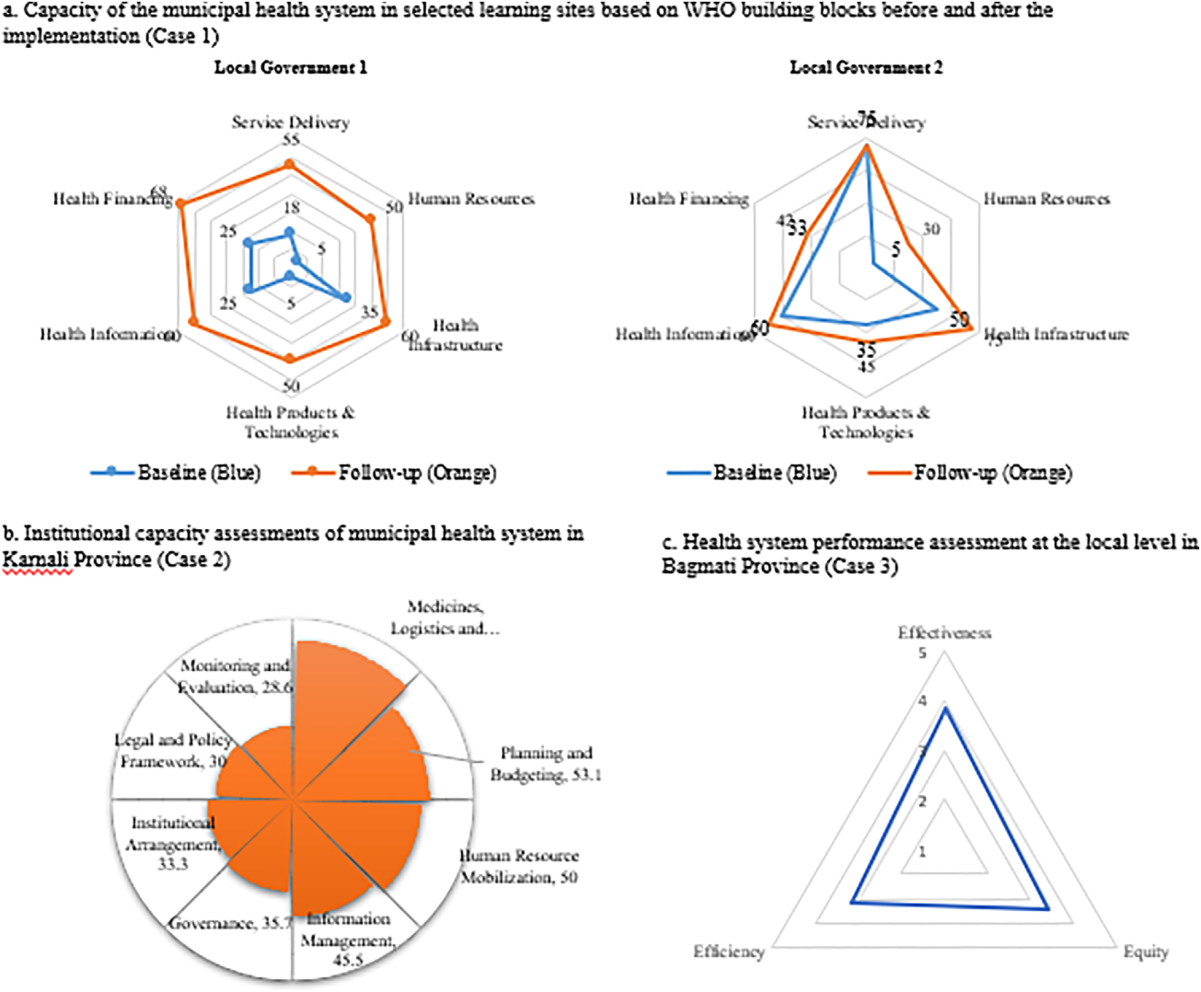
Health system capacity of sub‐national government using different tools and approaches. (a) Capacity of the municipal health systems in selected learning sites based on WHO building blocks before and after the implementation (Case 1). (b) Institutional capacity assessments of municipal health system in Karnali Province (Case 2). (c) Health system performance assessment at the local level in Bagmati Province (Case 3).

Human resources were identified as the weakest area, and targeted technical support was provided to strengthen them. The result demonstrates that well‐targeted and contextualized technical assistance can potentially strengthen municipal health systems’ capacity within a short period of time, particularly in a setting where baseline capacity is relatively weak [17, 18].

**Case 2**: Health Systems and Institutional Capacity Assessments were conducted in 2018 [19] as a participatory assessment in municipalities of Karnali Province that largely has rural settlements. The assessments were funded by the United States Government and used seven‐domain (0–100) scoring framework. As shown in Figure 2b, the median score across domains ranged from 28.6 to 62, suggesting the capacity gaps in achieving a desirable level of functionality. Functions that existed under the unitary system (such as medicine supply, planning and budgeting, and basic human resource management) tended to be stronger than newer governance functions, such as developing legal and policy frameworks or undertaking systematic monitoring and evaluation.

**Case 3**: The federal health system has also been strained and reshaped by the COVID‐19 pandemic, recurring disasters, and broader socioeconomic changes over the past decade. A recent mixed‐method study in a rural municipality of Bagmati Province in 2025 utilized 12 key informant interviews, one focus group discussion, and a survey of 166 households. The study applied an extended WHO framework to assess sub‐national health system performance, focusing on effectiveness, efficiency, and equity. Overall performance was moderate (3.48/5), with higher score for effectiveness (3.83/5) and equity (3.43/5) than for efficiency (3.17/5) [20]. In Figure 2c, health system effectiveness was constrained by geographic barriers (e.g., distance) and organizational issues (e.g., facility closures); equity between households due to limited insurance enrollment among poorer families; and efficiency due to staff shortages and intrahousehold decision‐making in care seeking.

The study also revealed deep‐rooted health worker demotivation, pervasive community distrust in health insurance schemes, poor decision‐making power among women, and persistent financial barriers for poor families. The study highlights social, geographic, organizational, and shock‐related determinants that shape sub‐national health system performance.

## 4 Opportunities and Challenges for Enhancing Capacity and Performance

These three case studies illustrate how Nepal's federalized health system has created both opportunities and challenges for building the capacity of local health systems and improving the performance of the sub‐national governments.

### 4.1 Opportunities

Nepal's federalization of the health system has created meaningful opportunities for stronger and more locally responsive systems. Targeted technical assistance has demonstrably strengthened system capacities across multiple health system components in a short timeframe, as evidenced in Case 1. Bottom‐up planning approaches, mandated under devolved roles, enhance community ownership, accountability, and context‐specific service delivery. At the same time, the gradual transfer of authority to LG fosters resilience and responsiveness to local needs.

### 4.2 Challenges

Despite the gains and promises, significant challenges persist. Capacity gaps remain and vary considerably across local levels. As highlighted in Case 2, the largest deficits are evident in newer governance functions, such as policy adoption and monitoring. Staff shortages and low motivation continue to threaten system performance and efficiency. In many of Nepal's municipalities, the sanctioned HRH staffing structure designates health assistants (HAs) or senior auxiliary health workers (AHW) as chiefs of health sections. These individuals, primarily trained in the delivery of basic health services, often face significant capacity constraints in meeting increasingly complex responsibilities, such as policy formulation, strategic planning, regulation, governance, and local health system monitoring. Urban–rural disparities, geographic barriers, and decision‐making power of women continue to constrain service reach, as evidenced in Case 3. When local leaders prioritize visible infrastructure over preventive and promotive services, such as immunization and maternal care, and devote limited attention to surveillance and emergency preparedness, concerns emerge regarding equity, efficiency, and overall system fragility.

## 5 Limitations

The three cases were purposively selected and applied different tools, scales, and units of analysis, so the findings are complementary snapshots rather than comparable measurements. Cases 1 and 2 rely on participatory self‐assessment, and Case 3 is confined to single municipality. These cases collectively reflect diverse scenarios and provide useful perspectives for measuring health system performance.

## 6 Conclusions

These three case studies demonstrate that federalization has facilitated the devolution of authority, which is critical but not sufficient to ensure equitable and efficient health systems. Achieving health goals requires stronger coordination among all three tiers of government to identify local needs and potentials and to mobilize targeted resources and efforts in areas of greatest need. Health systems assessment guided by a standardized framework can help to identify opportunities and address challenges. Reviewing health systems beyond WHO's building blocks is essential to recognize dynamics, interconnectivity, and contextual factors crucial for system strengthening. Sub‐national health systems with clearly defined functions, roles, and accountabilities are vital to fostering a resilient health system.

## Author Contributions

**Deepak Paudel**: conceptualization, methodology, formal analysis, writing – original draft, writing – review and editing, visualization. **Subheksha Adhikari**: methodology, formal analysis, visualization, writing – review and editing. **Ghanshyam Gautam**: writing – review and editing, methodology. **Yeshoda Aryal**: writing – review and editing. **Khim Bahadur Khadka**: conceptualization, writing – review and editing. **Sagar Prasad Ghimire**: writing – review and editing. **Sunita Dharel**: writing – review and editing. **Anil Bhattarai**: writing – review and editing. **Kimat Adhikari**: writing – review and editing.

## Funding

The authors have nothing to report.

## Ethics Statement

The data for this article are drawn from programmatic reports at the municipal level; no individual‐level information was collected except in Case 3, which received ethical approval from the Institutional Review Committee of Nobel College affiliated to Pokhara University, Nepal (Ref. 082/083/03). The process adhered to principles of informed consent, confidentiality, and voluntary participation.

## Conflicts of Interest

D.P. was employed by the USAID‐SSBH Project (Case 2), G.G. by the NHSSP (Case 1), and S.A. conducted Case 3 as part of her academic requirements. Authors, Y.A., K.B.K., S.P.G., S.D., and S.A., are affiliated with federal, provincial, and local governments. These roles have contributed to the work but may also have influenced the authors’ perspectives. Apart from these, the authors declare no other conflicts of interest.

## Data Availability

The data that support the findings of this study are available from the corresponding author upon reasonable request.
